# Multiple myeloma metabolism – a treasure trove of therapeutic targets?

**DOI:** 10.3389/fimmu.2022.897862

**Published:** 2022-08-22

**Authors:** Monica Roman-Trufero, Holger W. Auner, Claire M. Edwards

**Affiliations:** ^1^ Department of Immunology and Inflammation, Cancer Cell Protein Metabolism, The Hugh and Josseline Langmuir Centre for Myeloma Research, Centre for Haematology, Imperial College London, London, United Kingdom; ^2^ Nuffield Department of Surgical Sciences, University of Oxford, Oxford, United Kingdom; ^3^ Nuffield Department of Orthopaedics, Rheumatology and Musculoskeletal Sciences, University of Oxford, Oxford, United Kingdom

**Keywords:** plasma cell, multiple myeloma, metabolism, proteasome, cancer biology, proteostasis, bone marrow (BM) adipocytes

## Abstract

Multiple myeloma is an incurable cancer of plasma cells that is predominantly located in the bone marrow. Multiple myeloma cells are characterized by distinctive biological features that are intricately linked to their core function, the assembly and secretion of large amounts of antibodies, and their diverse interactions with the bone marrow microenvironment. Here, we provide a concise and introductory discussion of major metabolic hallmarks of plasma cells and myeloma cells, their roles in myeloma development and progression, and how they could be exploited for therapeutic purposes. We review the role of glucose consumption and catabolism, assess the dependency on glutamine to support key metabolic processes, and consider metabolic adaptations in drug-resistant myeloma cells. Finally, we examine the complex metabolic effects of proteasome inhibitors on myeloma cells and the extracellular matrix, and we explore the complex relationship between myeloma cells and bone marrow adipocytes.

## Introduction

Multiple myeloma (MM) is a cancer of plasma cells, the terminally differentiated effector cells of the humoral immune system whose main function is the provision of immunoglobulins. To fulfil this task, plasma cells dispose of a well-developed organellar apparatus that enables them to assemble and secrete large amounts of protein (1–3). Plasma cells, and MM cells, are also defined by their intricate interactions with the bone marrow microenvironment (4–6). Their highly specialized function and their distinct habitat shape the characteristic metabolic hallmarks of plasma cells and MM cells. Here, we aim to provide a concise introductory review of key metabolic features and adaptations of MM cells, discussing how their metabolic characteristics may contribute to resistance to anti-myeloma therapies, but also how they may be exploited for therapeutic purposes.

## Metabolic adaptations in non-malignant plasma cells

When naïve B cells are stimulated with antigen, they undergo a striking metamorphosis. Within days, their cytoplasm expands dramatically to accommodate a rapidly expanding endoplasmic reticulum and Golgi apparatus that push aside an increasingly heterochromatic nucleus (2). These morphologically distinct features reflect the highly specialized function of plasma cells, the synthesis and secretion of immunoglobulins. This complex process is resource-demanding and requires a broad range of metabolic adaptations, which are discussed comprehensively in several recently published reviews (7–9). Briefly, the bursts of proliferation and organelle biogenesis that occur during B cell activation and in germinal centers are supported by high levels of nutrient uptake, in particular of glucose and glutamine, which serve as major contributors to a diverse range of metabolic processes such as mitochondrial ATP generation, lipid synthesis, and ribonucleoside generation. Once plasma cell differentiation is complete, and the extensive secretory apparatus has been assembled, metabolic pathways are optimized again, and resources are pooled towards high levels of antibody synthesis and secretion, including extensive post-translational modifications. Glucose remains an important nutrient and is used primarily for antibody glycosylation and energy generation. In particular, the provision of pyruvate through glycolysis to enhance the ability to maximize ATP production through mitochondrial respiration and appears to be an important factor in promoting plasma cell longevity (10). While it remains to be established if and how plasma cells fine-tune lipid metabolism, it seems unlikely that this major part of cellular metabolism would not require any adaptations to high-level immunoglobulin production and maintenance of an extensive ER and Golgi network (7, 11). The complex metabolic demands of plasma cells to maintain energy homeostasis while assembling and secreting antibodies are also highlighted by the intricate role of autophagy to sustain long-lived humoral immunity and by their enhanced dependence on mitochondrial energy generation (10, 12, 13).

## Multiple myeloma dependency on glucose and glutamine

MM cells retain many of the metabolic features of plasma cells and further adapt them to meet the increased demands of malignant cells (14). This includes the heavy reliance of MM cells on glucose and glycolysis for energy generation (15–18). Clinically, the increase in glucose uptake underlies the use of 18F-Fluorodeoxyglucose (FDG)-positron emission tomography (PET) scanning to quantify and localize MM load in patients (19). MM cells also highly depend on glutamine, the most abundant amino acid in humans, not only as a protein building block but also as an anaplerotic substrate to replenish tricarboxylic acid (TCA) cycle components Glutamine depletion and inhibition of glutamine metabolism have already been shown to inhibit MM cell growth and to enhance sensitivity to anti-MM drugs (20–23). MM cells show high expression levels of glutaminase but not glutamine synthetase, a characteristic that renders the cells particularly dependent on extracellular glutamine. This dependency points to glutamine transporters as potentially useful therapeutic targets. While the expression of glutamine transporters such as LAT1, SNAT1 and ASCT2 increases with MM progression, the evidence so far indicates that MM cells mainly rely on ASCT2 for glutamine uptake. In particular, experiments combining the proteasome inhibitor, carfilzomib, with ASCT2 inhibitors have shown a synergic induction of proteotoxic stress and ROS generation suggesting a possible new therapeutic strategy (24, 25). Moreover, MM cells highly depend on mitochondrial energy generation (26–28). Thus, while the metabolic networks that support MM cell survival and proliferation and promote resistance to approved and investigational treatment approaches are only beginning to be understood, it is becoming increasingly clear that they may provide a plethora of novel opportunities for therapeutic interventions.

## Are proteasome inhibitors metabolic drugs?

Among the many anti-MM drugs that now form the therapeutic armamentarium that has dramatically improved outcomes for MM patients over the past 2 decades, proteasome inhibitors are a particularly intriguing class of drugs. Developed in the 1990 as experimental tools to better understand the function of the ubiquitin-proteasome system (UPS) in cell biology, they rapidly became the backbone of multiple regimens and are now widely used for the treatment of newly diagnosed and relapsed MM, in combination with essentially all other drug classes (29–31). Initially, their highly selective toxicity for MM cells was attributed to the high dependency of plasma cells on the UPS to clear so-called misfolded proteins (32–36), the toxic by-products of the complex and error-prone post-translational modification processes that secreted proteins undergo in the endoplasmic reticulum (ER). It has been widely assumed that proteasome inhibitors block the degradation of misfolded proteins, leading to their accumulation in the ER and overwhelming ER stress, and that this effect is particularly pronounced in MM cells, because their secretory load is exceptionally high (37). While ER stress may indeed represent one mechanism of action of proteasome inhibitors, it does not take into account the downstream effects of UPS inhibition on protein breakdown, namely the potentially lethal drop in intracellular amino acid availability and the wider metabolic consequences of such as effect (38–40). Not surprisingly, resistance to proteasome inhibition has been linked to hyperactivation of amino acid synthetic pathways. MM cell lines resistant to the proteasome inhibitor bortezomib show higher serine synthesis pathway (SSP) activity than bortezomib-sensitive cells. Phosphoglycerate dehydrogenase (PHGDH), the first rate-limiting enzyme in the SSP, was found to be significantly elevated in CD138+ cells derived from patients with relapsed MM, and high PHGDH expression conferred inferior survival. Mechanistically, PHGDH promotes proliferation and bortezomib resistance through increasing glutathione synthesis, thereby decreasing bortezomib-induced ROS generation, increasing effective protein folding, and promoting MM cell survival (41, 42). Metabolic reprogramming also supports regeneration of NAD(P)H and TCA cycle components to promote oxidative phosphorylation, and proteasome inhibitor-resistant cells contain structurally adapted mitochondria and have a different lipid content. These observations support the notion that proteasome inhibitors are complex metabolic drugs, and that targeting metabolic processes might offer novel therapeutic approaches to overcome proteasome inhibitor resistance (43, 44). To better understand the metabolic responses of MM cells to proteasome inhibition, we used a time-resolved integrated systems-level approach based on an *in vitro* model of proteasome inhibitor stress build-up and recovery that closely replicates typical clinical pharmacokinetics and antitumor responses in patients (45). These multi-omics time course studies allowed us to define the transcriptional, proteomic, and metabolic changes that occur in dying MM cells, and, perhaps more importantly, in MM cells that recovered from proteasome inhibition. These cells are relevant because they provide a faithful model for clinically proteasome inhibitor-resistant MM cells that ultimately cause relapse or progression. What we found was that surviving and resolving proteasome inhibitor-induced stress is a surprisingly protracted process during which MM cells undergo complex and dynamic changes of multiple metabolic pathways. These include a decrease in glucose uptake, a persistent drop in mitochondrial energy generation, enhanced lipid catabolism, and reduced intracellular levels of amino acids such as glutamine throughout and beyond immediate stress resolution. Importantly, the temporal patterns and functional connections of these waves of biological processes support a model in which at least some of the challenges that arise in surviving cells are directly linked to the mechanisms of stress resolution rather than the initial insult. As such, the cellular recovery processes generate potential therapeutic Achilles’ heels in the sense of dynamic trade-offs - temporarily increased vulnerabilities that are triggered specifically by the processes of stress resolution (46, 47). One of those vulnerabilities, in line with the proteasome inhibitor-induced prolonged depletion of intracellular amino acids, is that some MM cells depended on GCN2, the only kinase known to be activated by low amino acid levels (48–50), to recover from proteasome inhibition. Moreover, and highlighting the complex layers of metabolic regulation in MM cells in the wake of proteasome inhibition, we found GCN2 to be a major regulator of lipid metabolism during stress resolution. More recently, MM cell dependency on GCN2 has been linked to oncogenic MYC signaling (51), and observations that amino acid depletion sensitizes MM cells to proteasome inhibition by inducing compound mitochondria damage (52) provide further evidence for complex metabolic processes linked to proteasome inhibitors. We also found that drugs that target different aspects of mitochondrial function, such as the electron transport chain or mitochondrial translation, largely triggered a greater reduction in cell viability in recovering cells than in acutely stressed cells. These observations also raise the question if and how proteasome inhibitors or other drugs targeting cellular protein homeostasis might affect the myeloma microenvironment, in particular the formation of the extracellular matrix (ECM). We therefore applied a combination of materials science characterization techniques to an *in vitro* model of bone-like tissue formation by human bone-marrow derived mesenchymal stromal cells (hMSC). Intermittent and low-level inhibition of key UPS components, which triggered only very mild stress in the differentiating hMSCs, had surprisingly distinct effects on the bone-like material they formed, altering its stiffness and the amount of protein and crystalline mineral it contains, and affecting its micro- and ultra-structural organization (53). It remains to be established if such effects occur *in vivo*, and how they may affect MM cell behavior. However, the well-established crosstalk between physical tissue traits and cancer biology, for example the often-observed correlation of tissue stiffness with cancer aggressiveness and treatment resistance (54), raises the possibility that proteasome inhibitors might have effects on physical ECM characteristics that in turn alter MM cell responses to anti-cancer agents.

Additional evidence of the metabolic consequences of proteasome inhibitors is also provided by their interaction with mTOR, an integrator of environmental signals to regulate protein synthesis, autophagy, cell proliferation, growth and survival that plays a key role in MM and other cancers (55, 56). One of the signals that mTOR responds to is the availability of amino acids but, opposite to GCN2, mTOR activation by adequate amino acid availability promotes protein synthesis. Under conditions of amino acid deprivation, GCN2 has been shown to negatively regulate mTOR (57), indicating a functional interaction between both signaling pathways that is supported by observations that the discordant regulation of GCN2 and mTORC1 can adversely affect cell viability (58). It is therefore not surprising that experimental observations suggest that mTOR inhibition and proteosome inhibition may be synergistically toxic to MM cells (59, 60). However, clinical investigations into combining mTOR and proteasome inhibition have not resulted in major breakthroughs (61).

## Bone marrow adipocytes and myeloma

The bone marrow has a unique cellular composition, with cells such as bone marrow adipocytes, bone marrow stromal cells and immune cells ideally placed to interact with MM cells and modify their metabolic activity. Bone marrow stromal cells have been found to be the source of intercellular mitochondrial transfer, facilitated through tumor-derived tunneling nanotubes connecting stromal cells to MM cells (62). The consequence of this mitochondrial transfer was the promotion of oxidative phosphorylation, demonstrating MM metabolic plasticity driven by the bone microenvironment. There is increasing evidence to support the dysregulation of bone marrow adipocytes in MM, with reciprocal cross-talk between bone marrow adipocytes and MM cells not only promoting MM growth, chemoresistance and bone disease (63–67), but also inducing metabolic plasticity in both tumor cells and adipocytes. Bone marrow adipocytes are a major source of adiponectin, a key adipokine which we have previously shown to be decreased in MM, contributing to disease pathogenesis (68). More recently, we have shown that MM cells can downregulate adiponectin in bone marrow adipocytes, at least partly through TNFα signaling, providing a mechanism driving the hypoadiponectinemia associated with MM progression (69). MM cells have also been found to damage the mitochondria of adipocytes and preadipocytes, driving abnormal cytokine production, a senescence-like phenotype and disease progression (70, 71). While the role of lipid-uptake by MM cells and any functional consequences is unclear, recent studies demonstrate that myeloma-associated bone marrow adipocytes undergo lipolysis, associated with the uptake of secreted free fatty acids by adjacent MM cells (69, 70, 72). Obesity is one of the major risk factors for MM, with increased adiposity associated with the development of MM in preclinical models (73). The increase in adiposity has been associated with metabolic changes in MM cells, such as an increase in the metabolite acetyl-coA synthetase 2 (ACSS2) in MM cells, induced by adipocyte-secreted angiotensin II and driving MM growth *in vitro* and *in vivo* at least partly by enhancing the stability of the myeloma oncoprotein IRF4 (74). An increased understanding of the symbiotic relationship between tumor cells and bone cells can be gained by turning to solid tumor metastases such as breast and prostate cancer bone metastases, where commonalities in key mechanisms have led to similar approaches in combating the associated bone disease. There is increasing evidence to support the metabolic plasticity of tumor cells within the bone microenvironment (75). Our own recent observations used metabolomic profiling to identify elevation of the pentose phosphate pathway in prostate cancer cells, driven by interactions with bone cells. The rate-limiting enzyme of the pentose phosphate pathway, G6PD, was found to contribute to prostate cancer growth within the skeleton, representing a potential metabolic target for the treatment of bone metastasis (76).

As such, dissecting the metabolic cross-talk between MM cells and their surrounding microenvironment may reveal new therapeutic opportunities to disrupt the symbiotic relationship. Moreover, it will be fascinating, albeit far from straightforward, to explore if dietary interventions can have an impact on MM progression or the response to anti MM drugs.

**Figure 1:**
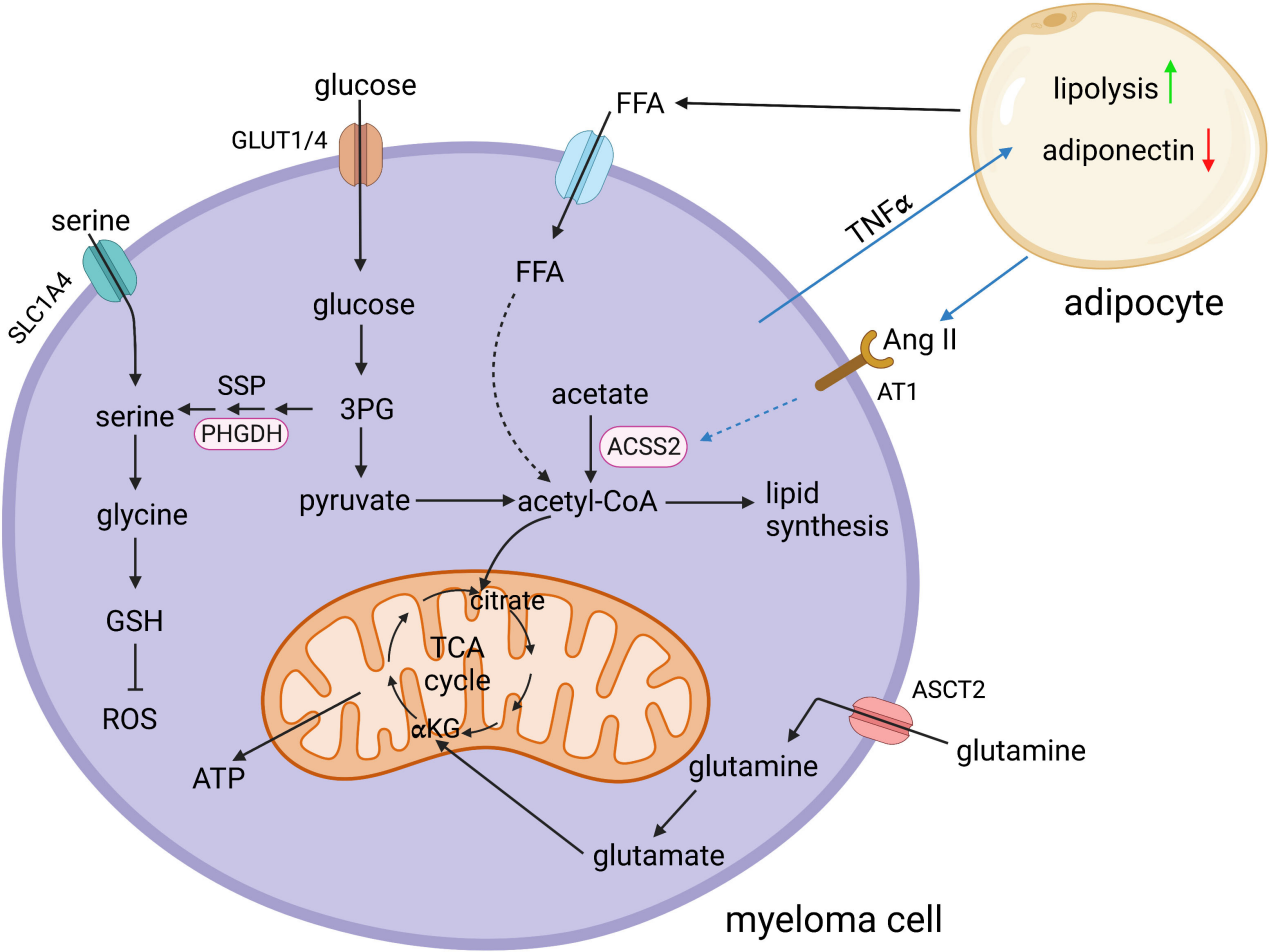
Schematic representation of selected key metabolic pathways in multiple myeloma cells and their interaction with bone marrow adipocytes. 3PG, 3-phosphoglycerate; ACSS2, acetyl-CoA synthetase 2; αKG, α-ketoglutarate; Ang II, angiotensin II; ASCT2, sodium-dependent neutral amino acid transporter type 2; AT1, angiotensin II receptor type 1; FFA, free fatty acids; GLUT1/4, glucose transporter 1/4; GSH, glutathione; PHGDH, phosphoglycerate dehydrogenase; ROS, reactive oxygen species; SLC1A4, solute carrier family 1 member 4; SSP, serine synthesis pathway; TCA cycle, tricarboxylic acid cycle; TNFα, tumor necrosis factor alpha. Created in BioRender.

In summary, a plethora of metabolic adaptations that occur in MM cells and in the myeloma microenvironment (**Figure 1**) may offer various approaches to delay the development or progression of MM, and to optimize treatment outcomes.

## Author contributions

MR-T, HA, and CE contributed to the conception, literature evaluation and writing of the review article. All authors contributed to the article revision, read, and approved the submitted version.

## Funding

MR-T and HA are funded by a Cancer Research UK Advanced Clinician Scientist Fellowship (C41494/A29035). CE is supported by Blood Cancer UK.

## Conflict of interest

The authors declare that the research was conducted in the absence of any commercial or financial relationships that could be construed as a potential conflict of interest.

## Publisher’s note

All claims expressed in this article are solely those of the authors and do not necessarily represent those of their affiliated organizations, or those of the publisher, the editors and the reviewers. Any product that may be evaluated in this article, or claim that may be made by its manufacturer, is not guaranteed or endorsed by the publisher.
